# An exploratory study of co-location as a factor in synchronous, collaborative medical informatics distance education

**DOI:** 10.1186/1756-0500-3-30

**Published:** 2010-02-02

**Authors:** Craig Locatis, Eta S Berner, Glenn Hammack, Steve Smith, Richard Maisiak, Michael Ackerman

**Affiliations:** 1 Office of High Performance Computing & Communications, National Library of Medicine, 8600 Rockville Pike, Bethesda, MD 20894, USA; 2 Department of Health Services Administration, School of Health Related Professions, University of Alabama at Birmingham, 1675 University Blvd. Birmingham, Alabama 35294, USA; 3 Department of Medical Student Services, School of Medicine, University of Alabama at Birmingham, 1530 3rd Ave. Birmingham, AL, USA; 4 Maisiak Associates, 5444 Grovers Ave. Scottsdale, Arizona 85254, USA

## Abstract

**Background:**

This study determined differences in learning, judgments of teaching and technology, and interaction when videoconferencing was used to deliver instruction on telemedicine to medical students in conditions where they were co-located and dispersed. A lecture on telemedicine was given by videoconference to medical students at a distant site. After a question and answer period, students were then given search problems on the topic and encouraged to collaborate. Half the students were randomly assigned to a co-located condition where they received the presentation and collaborated in a computer lab, and half were assigned to a dispersed condition where they were located in different rooms to receive the presentation and collaborate online using the videoconferencing technology. Students were observed in both conditions and they individually completed a test on presentation content and a rating scale about the quality of the teaching and the technology.

**Findings:**

There were no differences between the two groups in the learning outcomes or judgments about the teaching and technology, with the exception that more students in the dispersed condition felt more interaction was fostered. The level and patterns of interaction were very different in the two conditions and higher for dispersed students.

**Conclusions:**

Synchronous communication at a distance via videoconference may give sufficient sense of presence that the learning experience may be similar to that in actual classrooms, even when students are far apart. The technology may channel interaction in desirable ways.

## Background

Voice over IP and instant messaging companies, such as Skype and WebEx, are now offering two-way and multipoint videoconferencing services as more people have begun to get broadband access to their homes and workplace from fiber, cable, DSL, and other types of providers. The research undertaken here was done in anticipation that videoconferencing over IP will become more ubiquitous, increasingly practical, and more capable of facilitating much of the real time interaction possible with groups in classrooms. Indeed, most research on teaching with two way interactive video has been with groups, usually with a group at the teacher's classroom and a group at a remote site [1,2]. As the technology becomes more pervasive, it will be increasingly feasible for students to participate individually and be part of a virtual classroom. An important issue is how well such virtual classrooms can accommodate learning, especially if it involves collaboration. Consequently, this study examined learning outcomes, attitudes toward instruction and technology, and interaction when students participated in a collaborative distance learning experience by videoconference in contexts where they were co-located and where they were dispersed.

Distance learning programs can use asynchronous (email, web) and synchronous (videoconferencing) technologies [3] and may include "blended learning" approaches combining distance education with face-to-face instruction [4]. Three meta-analyses of distance education research are especially relevant to this study. One showed no significant differences between those taught at a distance and those taught in class, but sub-analyses of asynchronous distance learning and synchronous distance learning (defined as having students in class and others simultaneously participating remotely by video or audio conference) indicated small achievement advantages for asynchronous distance education and for classroom education when distance education was synchronous [1]. The authors were cautious about the finding, however, given the substantial heterogeneity and variability of the data. Another meta-analysis focusing only on video found students receiving distance instruction by television perform about the same as those in classrooms, but students in televised courses having two-way audio and video do better than those where communication is only one-way [2]. Finally, a meta-analysis of online learning studies found that achievement was higher when face-to-face instruction was blended with online learning [5].

The ability of students and teachers to see and hear each other in real time may increase sense of social presence and reduce transactional distance in communication, factors that are known to affect student satisfaction with distance learning [6-13]. Students at sites where conferences originate tend interact more and instructors concentrate more on students who are physically present [14]. Consequently, on site students tend to out perform distance ones and have better attitudes [1].

Videoconferencing technology can constrain dialog [9,14] and students at distant sites often feel more disconnected than those on site [12,15]. Detachment can be mitigated when students are not physically present at the origination site [16] and the overall level of interaction may influence student attitudes more than personal participation [17]. Students may appreciate hearing answers to questions others ask even though they do not ask questions themselves.

The closer media properties approximate in person conversation, the closer conversation style approximates face-to-face interaction. Consequently, factors such as high video quality, full duplex audio, and low latency can make videoconferencing conversations more personable [18,19]. The communication does not equate to face-to-face because of camera restrictions on the field of view, the need to use microphones, and other factors [12,19-21].

## Methods

Forty two medical student paid volunteers at the University of Alabama at Birmingham School of Medicine were randomly assigned to a learning activity involving the use of two-way interactive videoconferencing in a co-located condition and a dispersed one. In the co-located condition, students were physically together in a computer lab where they could interact with each other in person and with the remote instructor by videoconference. In the dispersed condition, students were physically separated in different rooms at the university and used desktop videoconferencing technology to interact both with the instructor and each other. Since the number of video sources that could be transmitted was limited, students in both conditions were exposed to the learning experience in small groups and the experience was repeated three times in each condition (groups of seven each in the dispersed condition and groups of ten, six, and five in the co-located condition).

**Figure 1:**
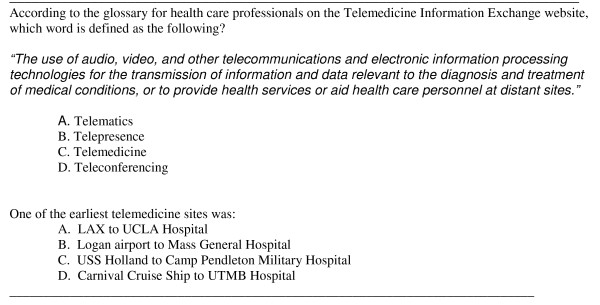
**Sample Test Questions**.

**Figure 2:**
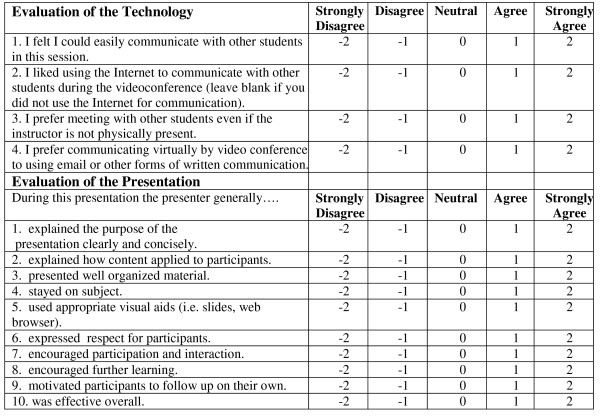
**Technology and Instruction Rating Forms**.

In both conditions, students were given a forty minute lecture on telemedicine by videoconference that was followed by a five minute question period and ten minute exercise searching a telemedicine web site. Students were told they could use any strategy to complete the exercise, but that they should try to work collaboratively. They took a short seventeen item multiple choice test on the lecture and search tasks and completed a scale with two sub-parts, one rating the instruction and one rating communicating with the technology. The instruction scale used modified items from a larger one developed at Stanford University that also included ratings of advising, mentoring and other aspects of teaching not relevant to this research [22]. One of the communication/technology questions about student communication via videoconferencing was only completed by dispersed students, since co-located students did not use the technology to communicate among themselves. Sample questions from the test appear in Figure 1. The instructional and communications technology rating scale appears in Figure 2. In addition, students were observed by two researchers; one physically present and one remotely in the co-located condition and two remotely in the dispersed condition. Remote observer video was blanked in both conditions.

## Results

Independent t-tests were performed to test for significant differences between conditions and among groups using SPSS. Item reliability analyses were also performed to determine internal consistency of the test and scales. Student ratings of instruction, responses to questions about communication and technology, and test scores were compared. The sub-scale for rating instruction was highly reliable (Cronbach's alpha = .88), while sub-scale rating communicating with the technology and the multiple choice test were moderately reliable (Cronbach's alpha = .50 and .42 respectively). The average ratings of instruction and communicating with the technology were generally high (Tables 1 &2) as were the multiple choice test scores (Table 3). Unpaired t-tests were performed on the test results, the communication technology ratings, and the ratings of instruction for both groups. There were no significant differences in the content learned as measured by the test (Table 3) or significant differences in the communication and technology ratings (Table 4). Only one of the ratings of instruction was significantly different (Table 5). Dispersed students felt the lecturer encouraged more interaction.

**Table 1:** Ratings of Instruction for Co-located and Dispersed Students

Item	Co-located Mean	SD	Dispersed Mean	SD
1. Purpose	1.20	.95	1.38	.59
2. Application	1.20	.77	1.10	.89
3. Organization	1.50	.61	1.52	.60
4. Stayed on Subject	1.50	.61	1.67	.48
5. Visual Aids	1.50	.61	1.38	.97
6. Respect	1.30	.80	1.57	.60
7. Interaction	.40	1.05	1.19	.87
8. Further Learning	.60	.99	.43	1.08
9. Motivation	.30	1.17	-.10	1.14
10. Overall	1.25	.79	1.24	.70

**Table 2:** Ratings of Communication/Technology for Co-located and Dispersed Students

Item	Co-located Mean	SD	Dispersed Mean	SD
1. Communicate with other students	.95	.71	.90	1.22
2. Using Internet to communicate	*	*	.95	.89
3. Prefer meeting with students	.79	1.18	.90	1.04
4. Prefer video to written communication	.58	1.02	.33	1.06

* Not rated by co-located students.

**Table 3:** Test Scores and T-Test Results of Co-located and Dispersed Students

			Mean	SD	Percent
Dispersed Multiple Choice Test			13.75	2.10	80%
Co-located Multiple Choice Test			14.10	1.34	82%

	t	df	Significance (2-tailed)		Standard Error Difference

Co-located - Dispersed	-.63	39.00	.53		.55

(Maximum score = 17).

**Table 4:** Communication/Technology Ratings T-Test Results for Co-located and Dispersed Students

Item	t	df	Significance (2-tailed)	Standard Error Difference
1. Communicate with other students	.13	38.00	.89	.32
2. Using Internet to communicate	*	*	*	*
3. Prefer meeting with students	-.33	38.00	.75	.35
4. Prefer video to written communication	74	38.00	.46	.33

* Not rated by co-located students and not analyzed.

**Table 5:** Instruction Ratings T-Test Results for Co-located and Dispersed Students

Item	t	df	Significance (2-tailed)	Standard Error Difference
1. Purpose	-.74	39.00	.47	.25
2. Application	.40	39.00	.69	.26
3. Organization	-.13	39.00	.90	.19
4. Stayed on Subject	-.98	39.00	.34	.17
5. Visual Aids	.47	39.00	.34	.17
6. Respect	-1.23	39.00	.22	.22
7. Interaction	-2.63	39.00	.01*	.30
8. Further Learning	.53	39.00	.60	.32
9. Motivation	1.10	39.00	.28	.36
10. Overall	.05	39.00	.96	.23

The number of questions asked the lecturer ranged from two in one co-located session to six in one dispersed session with four questions asked in other sessions. Since interactions initiated by individual students in the collaborative exercise were not recorded, they could not be compared statistically. There were fourteen interactions in co-located sessions and thirty nine interactions in the dispersed. Co-located interactions may have been undercounted somewhat due to the difficulty of monitoring students spread throughout the computer lab, but there was still more interaction in the dispersed condition. The kind of interaction varied. In one co-located session, students agreed to divide the search questions, work independently, and share results. Interactions in the co-located condition were limited to those in close physical proximity, usually students sitting besides each other. In contrast, videoconferencing in the dispersed condition included everyone in the conversation, even if they did not say anything.

## Discussion

The absence of students at the instructor's site eliminated the instructor focusing attention on those physically present. The fact that there were no performance differences and only one significantly different rating between the co-located and dispersed students indicates that the real time virtual interaction that videoconferencing affords had no detrimental impact on performance or students ratings and may have positively affected participation and interaction.

The students in the dispersed condition rated the lecturer's encouraging interaction significantly higher than those who were co-located and they interacted more because the technology extended access to conversation to all participants, not just some one physically nearby. It is likely that these higher levels of interaction made the dispersed students more inclined to give the lecturer higher marks for encouraging interactivity since ratings of instruction were not done until the very end of the session, after students collaborated and took the test. Consequently, it can be viewed more as an assessment of the entire learning experience, not just the lecture.

It is uncertain whether the interactivity effects observed in this study would carry over for larger groups of students, especially if dispersed. There are videoconferencing technologies that can connect more end points and those conducting videoconferences routinely generally acknowledge more end points become harder to manage. On the other hand, interaction also is harder to manage when larger classes meet in person. It is possible to control for group size to some extent in videoconferencing by having dispersed students access different, smaller videoconference sessions, rather than one large one, the virtual equivalent to breaking a large class into small groups.

It is also uncertain if the interactivity effects observed would occur if students had to perform their collaborative tasks differently. In the study, students worked collaboratively from print outs independently using software on their own machines. If dispersed students had to take turns using an application on a single desktop and pass control to each other, which also can be done with some online collaboration tools, the mechanics of working together may have been more cumbersome. But it is not always necessary or desirable that students share the same application while they work together, whether they are co-located or not.

It became apparent in the course of the study that there were certain transactional and distance factors affecting interaction even when students were co-located. Consider a group of people standing or sitting together at a reception or party. They will hear their own conversation and perhaps parts of others nearby, but will unlikely hear conversations across the room. To include others in their conversation they might have to raise their voices, make attention getting noises, or relocate. There are, of course, social, cultural, and language differences that inject "distance" into any communication. Distance factors affect communication even when people occupy the same physical space. With the exception of the session where a student approached others and suggested they divide the assignment, no effort was made to move or reconfigure seating in the co-located sessions to better accommodate collaboration. Students partnered with the person next to them.

## Conclusion

Synchronous communication at a distance via videoconference may give sufficient sense of presence that the learning experience may be similar to that in actual classrooms, even when students are far apart and have to work collaboratively. In asynchronous online learning students are separated by time and place while in synchronous online learning they are only separated by location. Students are still physically separate from each other, more or less, by location, even when they occupy the same classroom, and this separation can impact communication as much as if they were separated at a greater distance.

This experiment suggests that videoconferencing can mimic many conditions in normal classrooms when students are individually apart and might potentially provide a more inclusive and accessible communication environment. It may be a useful method to bring students together during preceptorships and other phases of education where they learn remotely and as a method to provide real time continuing education experiences to practitioners not wanting to travel. Bringing individual students from diverse locations virtually by videoconference may be as interactive or more interactive than bringing them physically together in classrooms. One area for future research is whether bringing students together virtually by videoconference can be combined with asynchronous distance education as a way to implement a new form of blended learning.

## Permissions

This research was approved by the Institutional Review Boards of the University of Alabama at Birmingham and the National Institutes of Health.

## Competing interests

The authors declare that they have no competing interests.

## Authors' contributions

CL Conceived the general research plan, worked on developing the measuring instruments, assisted in implementing the technology and wrote the original manuscript. EB Developed the detailed research plan, worked on developing the measuring instruments, was lead in implementing the technology and collaboration tasks, assisted with data collection and writing the manuscript. GH Provided the lesson content and was the distance education instructor. SS Recruited subjects and assisted in collecting data and writing the manuscript. RM Provided statistical consultation and performed the statistical analysis. MA Assisted in the development of the general research plan, the writing of the original manuscript, and editing and revising later versions. All authors have read and approve the final manuscript.
